# Structural plasticity of the bilateral hippocampus in glioma patients

**DOI:** 10.18632/aging.103212

**Published:** 2020-06-05

**Authors:** Taoyang Yuan, Jianyou Ying, Zhentao Zuo, Songbai Gui, Zhixian Gao, Guilin Li, Yazhuo Zhang, Chuzhong Li

**Affiliations:** 1 Beijing Neurosurgical Institute, Capital Medical University, Beijing, China; 2 Department of Neurosurgery, Beijing Tiantan Hospital, Capital Medical University, Beijing, China; 3 Beijing Institute for Brain Disorders Brain Tumor Center, Beijing, China; 4 China National Clinical Research Center for Neurological Diseases, Beijing, China; 5 State Key Laboratory of Brain and Cognitive Science, Institute of Biophysics, Chinese Academy of Sciences, Beijing, China

**Keywords:** gliomas, structural MRI analysis, dynamic amplitude of low-frequency fluctuation (dALFF), hippocampus, plasticity

## Abstract

This study investigates the structural plasticity and neuronal reaction of the hippocampus in glioma patient pre-surgery. Ninety-nine glioma patients without bilateral hippocampus involvement (low-grade, n=52; high-grade, n=47) and 80 healthy controls with 3D T1 images and resting-fMRI were included. Hippocampal volume and dynamic amplitude of low-frequency fluctuation (dALFF) were analyzed among groups. Relationships between hippocampal volume and clinical characteristics were assessed. We observed remote hippocampal volume increases in low- and high-grade glioma and a greater response of the ipsilateral hippocampus than the contralesional hippocampus. The bilateral hippocampal dALFF was significantly increased in high-grade glioma. Tumor-associated epilepsy and the IDH-1 mutation did not affect hippocampal volume in glioma patients. No significant relationship between hippocampal volume and age was observed in high-grade glioma. The Kaplan-Meier curve and log-rank test revealed that large hippocampal volume was associated with shorter overall survival (OS) compared with small hippocampal volume (p=0.007). Multivariate Cox regression analysis revealed that large hippocampal volume was an independent predictor of unfavorable OS (HR=3.597, 95% CI: 1.160-11.153, p=0.027) in high-grade glioma. Our findings suggest that the hippocampus has a remarkable degree of plasticity in response to pathological stimulation of glioma and that the hippocampal reaction to glioma may be related to tumor malignancy.

## INTRODUCTION

The hippocampus is one of the few regions of the adult brain that contains neural stem cells and can continue to generate neurons throughout life in humans and animals [1–4]. Primary neurogenesis in the adult hippocampus is dynamic and involves a selective response to pathophysiological stimuli [4, 5]. Newborn neurons usually migrate and integrate into the established neural circuitry to improve cognitive function or to resist diseases such as brain trauma and depressive disorders [5, 6]. In the human brain, neuroimaging studies have demonstrated that the hippocampus has a remarkable degree of plasticity in response to a variety of trainings, experiences, and psychiatric disorders. For example, human hippocampal volume has been shown to increase in response to aerobic exercise and to return to baseline after an additional six weeks without aerobic exercise [7]. In major depressive disorder (MDD), hippocampal volume alterations have been suggested to represent a potentially useful biomarker for the development or treatment of MDD; patients with more depressive episodes show greater hippocampal volume loss, and antidepressant treatment or electroconvulsive therapy can increase hippocampal volume [8–10]. In Alzheimer's disease, hippocampal atrophy is a core biomarker and T1-weighted magnetic resonance imaging (MRI) measurements of the hippocampus are a powerful tool for monitoring progression and evaluating therapeutic effects [11, 12]. Similar hippocampal volume alteration has also been detected by histological studies in animals [13–15].

Gliomas are the most prevalent primary intracranial tumors, their outstanding characteristic are the infiltration and destruction of surrounding and remote brain tissues, and invading tumor cells migrate to remote brain regions [16]. To assess tumor malignancy, gliomas can be subclassified into 4 grades (grade I, grade II, grade III and grade IV) based on the World Health Organization (WHO) histopathological criteria [17]. High-grade gliomas (HGG), which includes grades III and IV, is believed to have a faster growth rate, higher recurrence, poorer prognosis, and shorter survival time than low-grade gliomas (LGG), which includes grades I and II [18, 19]. As defined by the WHO classification in 2016, grades II, III, and IV are further subdivided into isocitrate dehydrogenase (IDH) mutant and IDH wildtype [20]. IDH mutation is an early event in glioma genesis and has significant implications for glioma progression and tumor behavior [21]. A previous study found that tumor cells invaded areas remote to the primary glioma site along white matter fibers based on immunohistochemistry for IDH1-R132H in high-grade glioma [22].

Multiple studies have reported that hippocampal volume significantly decreases in glioma patients treated with standard chemoradiation compared to healthy controls [23, 24]. Volume loss in brain regions remote to the site of glioma before surgery has also been found using modern imaging methods. For instance, focal glioma in the cerebral cortex leads to volumetric and diffusion alterations in distant subcortical areas, such as the caudate nucleus, putamen, and thalamus, as well as microstructural impairment of extratumoral whole-brain normal-appearing white matter [25, 26]. Recently, one study reported that the hippocampus appears to be a brain region that is less prone than other regions to tumor invasion [27]. In addition, secondary neuronal reactions in the remote hippocampus were detected in glioma rat model by positron emission tomography (PET) [28]. However, to date, no studies have observed structural alterations and neuronal reactions in the hippocampus distant to tumor in patients with glioma prior to surgery.

## RESULTS

### Clinical and demographic characteristics

A total of 99 glioma patients with no left or right hippocampus involvement (LGG, n = 52, including left LGG n = 25 and right LGG n = 27; HGG, n = 47, including left HGG n = 25 and right HGG n = 22) and 80 HCs were used in the analysis. The detailed demographic and clinical characteristics of the subjects are summarized in Table 1. All patients underwent surgical treatment, and the postoperative pathological diagnosis was glioma. The TIV in LGG patients (1473 ± 106 cm^3^) and HGG patients (1474 ± 116 cm^3^) was significantly increased compared to that in HCs (1387 ± 139 cm^3^) (one-way ANOVA, p < 0.0001). No differences in age (one-way ANOVA, p = 0.052), education (one-way ANOVA, p = 0.057), or sex (χ2 test, p = 0.057) were found among the three groups. 22 patients in the LGG group and 19 patients in the HGG group presented with TAE. In the HGG group, pathological information regarding the IDH1 mutation status was collected (IDH1_WT_, n = 23; IDH1_M_, n = 24), and all patients had received standard-of-care treatment, which included surgical resection followed by radiation therapy as well as concurrent and adjuvant temozolomide. In the LGG group, pathological information on the IDH1 mutation status had not been collected in 6 patients. Overall survival (OS), which was defined as the time between initial surgical treatment and death caused by the tumor or last follow-up, was obtained and the median OS was 24.0 months (range, 5.0–30.0 months) in 47 high-grade glioma patients. In addition, 71 meningioma patients with no left or right hippocampus involvement and 73 healthy controls were recruited in our study. The detailed clinical information of these subjects is summarized in Table 2. No differences in age (two-sample t test, p = 0.31), education (two-sample t test, p = 0.06), or sex (χ2 test, p = 0.77) were found between the two groups.

**Table 1:** Demographic and clinical characteristics among glioma patient groups and healthy controls (HCs).

**Variable**	**Low-grade glioma group**	**High-grade glioma group**	**Healthy controls**	**P value**
Numbers	52	47	80	NA
Age (mean ± SD), y	40.2 ± 9.6	45.8 ± 12.9	42.4 ± 11.5	p = 0.052^a^
Sex ratio, F/M, n	21/31	23/24	49/31	P = 0.057^b^
TIV (cm^3^)	1473 ± 106	1474 ± 116	1387 ± 139	P < 0.001^a^
Education (mean ± SD)	12.5 ± 3.7	11.9 ± 3.6	13.5 ± 3.9	p = 0.057^a^
Tumor grade, I/II, III/IV, n	0/52	22/25	NA	NA
Contrast enhanced volume (mean ± SD), cm^3^	0.2 ± 0.9	29.4 ± 20.3	NA	NA
Epilepsy, n	22	19	NA	NA
Tumor side (left hemisphere/right hemisphere), n	25/27	25/22	NA	NA
IDH1_WT_/IDH1_M_, n	4/42	23/24	NA	NA

^a^ represent one-way ANOVA. ^b^ represent χ2 test.

Abbreviations: TIV, total intracranial volume; IDH1_M_, IDH1 mutation; IDH1_WT_, IDH1 wild type.

**Table 2:** Demographic and clinical characteristics between meningioma patients and healthy controls (HCs).

**Variable**	**Meningioma group**	**Healthy controls**	**P value**
Numbers	71	73	NA
Age (mean ± SD), y	50.3 ± 11.2	48.5 ± 9.4	p = 0.31^a^
Sex ratio, F/M, n	46/25	49/24	P = 0.77^b^
Handedness, R/A/L, n	66/2/3	69/1/3	NA
Education (mean ± SD)	11.5 ± 3.9	12.7 ± 3.8	p = 0.06^a^

^a^ represent two-sample t test. ^b^ represent χ2 test.

### GMV increase in ipsilateral hippocampus in glioma patients by VBM analysis

**Figure 1:**
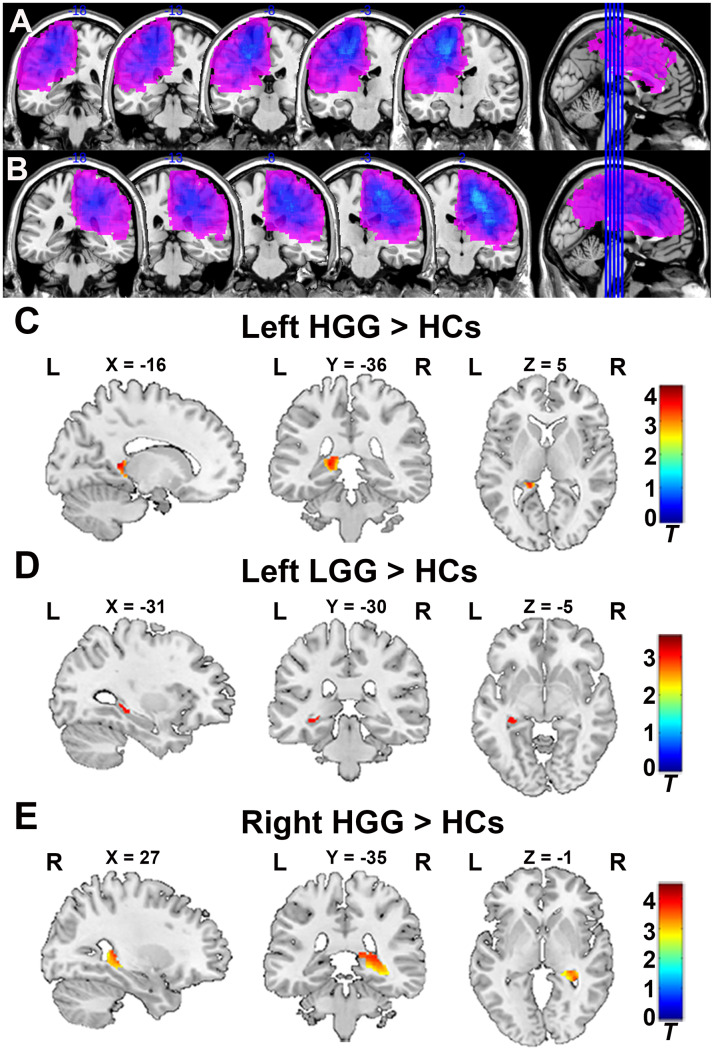
**Tumor overlapping map across glioma patients and VBM analysis.** (**A**) All tumor masks in left hemisphere gliomas (including left LGG and HGG) overlapped in the Ch2bet template. (**B**) All tumor masks in right hemisphere gliomas (including right LGG and HGG) overlapped in the Ch2bet template. VBM analysis showing GMV increases in the ipsilateral hippocampus in the left HGG group (**C**), left LGG group (**D**), and right HGG group (**E**) compared to HCs (FDR-corrected, p < 0.05, cluster size > 20). L: left hemisphere, R: left hemisphere.

To verify glioma patients with no left or right hippocampus involvement, the tumor overlapping maps for left and right hemispheric glioma are displayed in Figure 1A, 1B. As shown in Figure 1C, 1E, the GMV in the ipsilateral hippocampus significantly increased in the left and right HGG groups compared to HCs (FDR-corrected cluster level p < 0.05, Supplementary Table 1). Similarly, as shown in Figure 1D, the GMV in the ipsilateral hippocampus was also significantly increased in the left LGG group compared to HCs (FDR-corrected cluster level p < 0.05, Supplementary Table 1). No increase in the GMV in the contralesional hippocampus was found in any patient groups compare to HCs.

### Hippocampal volume increases in glioma patients with ROI-based analysis

**Figure 2:**
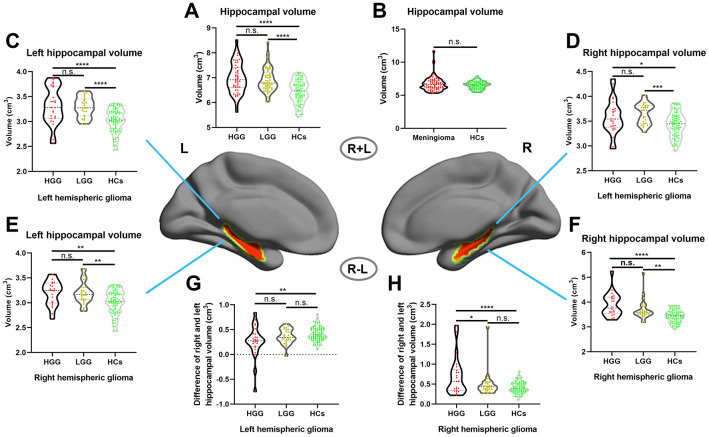
**Increased hippocampal volume in glioma patients with ROI-based analysis.** (**A**) Hippocampal volume significantly increased in both the HGG and LGG groups compared to HCs (one-way ANOVA with Bonferroni correction, **** p < 0.0001, n.s. represents no significance). (**B**) No significant difference in hippocampal volume between meningioma patients and HCs (two-sample t test, p = 0.24). (**C**, **D**) Right and left hippocampal volume significantly increased in left hemispheric glioma (including left LGG and left HGG) compared to HCs (one-way ANOVA with Bonferroni correction, * p < 0.05, *** p < 0.001, **** p < 0.0001). (**E**, **F**) Right and left hippocampal volume significantly increased in right hemispheric glioma (including right LGG and right HGG) compared to HCs (one-way ANOVA with Bonferroni correction, ** p < 0.01, **** p < 0.0001). (**G**, **H**) For right and left hemispheric high-grade glioma patients, there was a significant increase in the ipsilateral hippocampal volume relative to the contralesional hippocampal volume in the HGG group compared to the LGG and HCs groups (one-way ANOVA with Bonferroni correction, ** p < 0.01, **** p < 0.0001). L: left hemisphere, R: left hemisphere**.**

First, we assessed the alterations of hippocampal volume (including left and right hippocampal volume) in gliomas of different grades, and the results showed that hippocampal volume was significantly increased in both the HGG and LGG groups compared with the HCs, as shown in Figure 2A (one-way ANOVA with Bonferroni correction, p < 0.0001). In addition, we investigated whether this phenomenon existed in meningioma patients, and the results revealed no significant difference in hippocampal volume between meningioma patients and HCs, as shown in Figure 2B (two-sample t test, p = 0.24). Next, we detected alterations in the ipsilateral and contralesional hippocampal volume in unilateral glioma and found that both the left and right hippocampal volume significantly increased in the LGG and HGG groups compared to HCs, as shown in Figure 2C–2F (one-way ANOVA with Bonferroni correction, p < 0.05). To analyze detailed effect of gliomas on the ipsilateral and contralesional hippocampal volume, the difference between the right and left hippocampal volume was calculated in all subjects. Interestingly, as shown in Figure 2G, 2H, we found that left and right hemispheric high-grade gliomas caused a significant increase in the ipsilateral hippocampal volume relative to the contralesional hippocampal volume in the HGG group compared to the LGG group and HCs.

### The impact of TAE and IDH-1 mutation status on hippocampal volume in glioma patients

**Figure 3:**
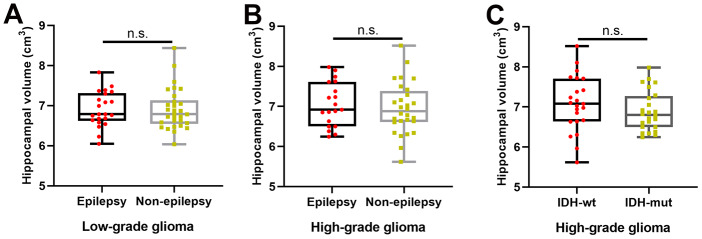
**The effects of tumor-associated epilepsy and IDH-1 mutation status on hippocampal volume**. (**A**, **B**) No significant difference in hippocampal volume between patients with and without tumor-associated epilepsy in the LGG (two-sample t test, p = 0.98) and HGG groups (two-sample t test, p = 0.61). (**C**) There was no significant difference in hippocampal volume between patients with IDH-1 mutations and patients with IDH-1 wild-type mutations in the HGG group (two-sample t test, p = 0.29). L: left hemisphere, R: left hemisphere.

As shown in Figure 3A, 3B, there was no significant difference in hippocampal volume between patients with and without TAE in the LGG (two-sample t test, p = 0.98) and HGG groups (two-sample t test, p = 0.61). Similarly, as shown in Figure 3C, we also found no significant difference in hippocampal volume between patients with IDH-1 mutation and patients with IDH-1 wild-type in HGG group (two-sample t test, p = 0.29).

### Increased hippocampal activity in high-grade glioma patients

**Figure 4:**
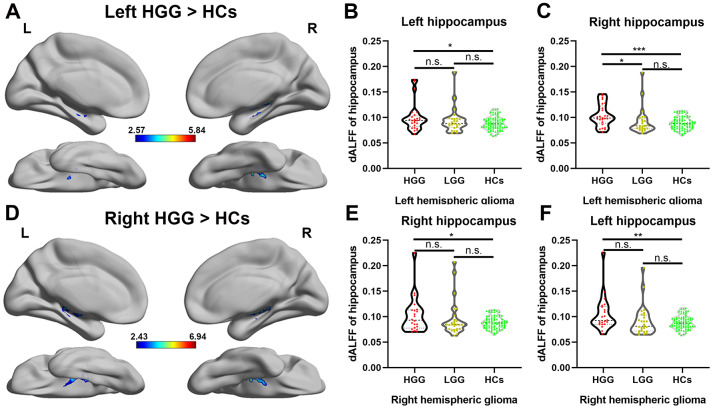
**Alteration of the dALFF in high-grade glioma patients.** (**A**) The left HGG group showed significantly increased temporal variability of the dALFF in the bilateral hippocampus compared to HCs using a two-sample t-test (FDR-corrected cluster level p < 0.05, cluster size > 10). (**B**, **C**) For left hemispheric high-grade glioma patients, a significant increase in the dALFF was observed in both the entire left and right hippocampi in the HGG group compared to HCs (one-way ANOVA with Bonferroni correction, * p < 0.05, *** p < 0.001). (**D**) The right HGG group showed significantly increased temporal variability of the dALFF in the bilateral hippocampus compared to HCs using a two-sample t-test (FDR-corrected cluster level p < 0.05, cluster size > 10). (**E**, **F**) For right hemispheric glioma, a significant increase in the dALFF was observed in both the entire left and right hippocampi in the HGG group compared to HCs (one-way ANOVA with Bonferroni correction, * p < 0.05, ** p < 0.01).

Differences in the dALFF were quantified for each voxel in the hippocampus between the patient groups and HCs, as shown in Figure 4A, 4D, left and right HGG groups showed significantly increased dALFF in bilateral hippocampus compared to HCs using two sample t-test (FDR-corrected cluster level p < 0.05). However, compared to HCs, in the LGG group, there was no significant increase in the dALFF in the bilateral hippocampus. Furthermore, we extracted the value of dALFF in all subjects and detected the alterations of neuronal activity in entire hippocampus on each side among the LGG, HGG, and HCs. As shown in Figure 4B, 4C, 4E, 4F, a significant increase in the dALFF in both the left and right hippocampus weas observed in the HGG group compared to HCs (one-way ANOVA with Bonferroni correction, p < 0.05).

### Correlational analysis between hippocampal volume and clinical features

**Figure 5:**
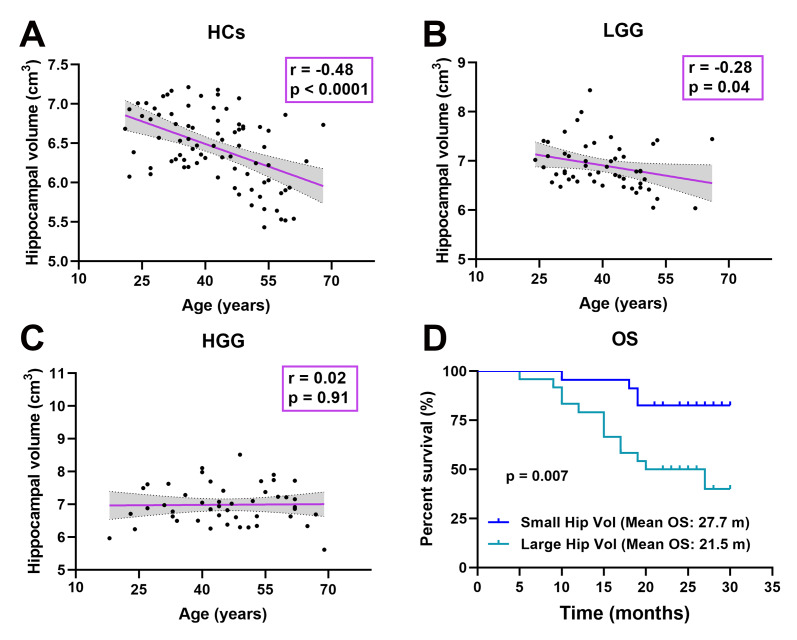
**Correlation analysis.** (**A**) Significant negative correlation between age and hippocampal volume in HCs (r = -0.48, p < 0.0001). (**B**) Significant negative correlation between age and hippocampal volume in the LGG group (r = -0.28, p = 0.04). (**C**) There was no significant correlation between age and hippocampal volume in the HGG group (r = 0.02, p = 0.91). (**D**) Kaplan-Meier curves and log rank tests showed that a large hippocampal volume (≥ 6.92 cm^3^, n = 24) (mean OS: 21.5 months, 95% CI: 18.1-24.9 months) was associated with a shorter OS than a small hippocampal volume (< 6.92 cm^3^, n = 23) (mean OS: 27.7 months, 95% CI: 25.5-29.8 months) in high-grade glioma patients (n = 47, p = 0.007).

In all subjects, the relationship was detected between age and hippocampal volume. Interestingly, as shown in Figure 5A–5C, the relationship significant decrease in the LGG group (r = -0.28, p = 0.04) and disappeared in the HGG group (r = 0.02, p = 0.91) compared to HCs (r = -0.48, p < 0.0001). In the HGG group, we investigated the relationship between hippocampal volume and OS. The median hippocampal volume was 6.92 cm^3^; thus, we classified the high-grade glioma patients into a large hippocampal volume group (≥ 6.92 cm^3^, n = 24) and a small hippocampal volume group (< 6.92 cm^3^, n = 23). Kaplan-Meier curves and log rank tests were used to evaluate the correlations between CHGMV and OS, and the results indicated that a large hippocampal volume (mean OS: 21.5 months, 95% CI: 18.1-24.9 months) was associated with a shorter OS than small hippocampal volume (mean OS: 27.7 months, 95% CI: 25.5-29.8 months), as shown in Figure 5D (p = 0.007). To further determine whether hippocampal volume is an independent predictor of OS in high-grade gliomas, we performed Cox proportional hazards regression analyses. First, a univariate Cox proportional hazards regression analysis was performed to evaluate the factors affecting OS in patients such as hippocampal volume, histological grade (III, IV), age, contrast-enhance volume, IDH1 status (see Table 3). Second, all factors with a P value < 0.1 were further evaluated by the forward stepwise multivariate Cox proportional hazards regression model, which suggested that a large hippocampal volume was an independent factor for poor OS (HR = 3.597, 95% CI: 1.160-11.153, p = 0.027), even after adjusting for the histological grade (HR = 19.796, 95% CI: 2.606-150.376, p = 0.004) in high-grade glioma patients (see Table 3).

**Table 3:** Univariate and multivariate cox regression analysis of variables to predict OS.

**Variables**	**Univariate analysis**			**Multivariate analysis**		
	**B**	**HR (95% CI)**	**P value**	**B**	**HR (95% CI)**	**P value**
Age, year	0.046	1.047 (1.006-1.090)	0.023	-	-	-
Hippocampal volume (large ≥ 6.92, small < 6.92)	1.400	4.056 (1.319-12.474)	0.015	1.280	3.597 (1.160-11.153)	0.027
Contrast enhanced volume, cm^3^	0.017	1.017 (0.997-1.039)	0.103	-	-	-
histological grade (III/IV)	3.051	21.131 (2.791-159.982)	0.003	2.985	19.796 (2.606-150.376)	0.004
IDH1 (wild type, mutation)	-1.310	0.27 (0.095-0.768)	0.014	-	-	-

## DISCUSSION

The hippocampus has a remarkable degree of plasticity in response to a variety of pathophysiological stimuli. Furthermore, hippocampal volume is usually recognized as a valuable imaging biomarker for the diagnosis, progression or treatment of multiple psychiatric disorders. In the current study, we investigated volume alterations and neuronal activity of the hippocampus in glioma patients with no left or right hippocampus involvement before surgery using structural MRI and resting-fMRI for the first time. Our results showed that (a) both low- and high-grade gliomas, especially high-grade glioma, could cause an increase in remote hippocampal volume and the ipsilateral hippocampus had a more prominent response than the contralesional hippocampus; (b) dynamic regional neuronal activity in the left and right hippocampus was significantly increased in high-grade glioma; (c) TAE and IDH-1 mutation status did not affect the hippocampal volume in low- and high-grade gliomas; (d) glioma destroyed the relationship between hippocampal volume and age; and (e) a large hippocampal volume was associated with a shorter OS than small hippocampal volume in high-grade glioma.

VBM, a fully automated computerized quantitative MRI analysis technique, enables voxel-wise statistical comparison of the GMV or WMV between groups in brain anatomy [29]. Using VBM, we found a significant increase in the GMV in the ipsilateral hippocampus in both the left and right HGG groups, as well as in the left LGG group. Considering that VBM is more sensitive to localized changes that affect the same location (or voxels) in every subject, we performed ROI-based hippocampal volume analysis which can reveal total volume alterations in the hippocampus. Our results showed that the ipsilateral hippocampal volume significantly increased in both the HGG and LGG groups, and that the contralesional hippocampal volume also significantly increased in both the HGG and LGG groups compared to HCs.

The hippocampus is one of the few regions in the adult brain that contains neural stem cells, which can induce a neurogenesis response to pathophysiological stimuli, such as training, trauma, and ischemia [4, 30]. Multiple studies, including a combined MRI and histology study in animals, have demonstrated that neurogenesis is the main reason for the GMV increase in the hippocampal response to pathophysiological stimuli [14, 15]. In the current study, the mechanisms underlying the remarkable increase in the bilateral hippocampal volume in glioma patients with no left or right hippocampus involvement may be due to obvious neurogenesis in the hippocampus under the stimulation of the pathological state of glioma. This neurogenesis may repair the structural and functional damage caused by the tumor. Interestingly, a significant increase in the ipsilateral hippocampal volume relative to the contralesional hippocampal volume was observed in glioma patients. This result may be due to the fact that the hemisphere where the tumor is located has more serious damage relative to the contralateral hemisphere, resulting in stronger neurogenesis of the ipsilateral hippocampus. In addition, this phenomenon of the study was retested in patients with intracranial meningiomas and we did not find hippocampal volume increases in meningioma patient group, which may be due to the fact that meningioma arises from the dura mater, and the main effect on brain structure is pushing and pressing rather than damage [31]. The detailed physiological mechanism of hippocampal volume enlargement in glioma patients requires further histological study. In healthy populations, brain volume in different patients is variable, so some studies calculated relative volume. In our study, we found that the TIV in the LGG and HGG groups was significantly increased compared to that in healthy controls. This result may suggest that infiltrative and swelling growth of the tumor increases brain volume in patients with glioma. Considering that enhancing TIV may affect the accuracy of the relative hippocampal volume in patients with glioma, the hippocampus volume relative to the TIV was not calculated in our study.

In the current study, neuronal activity of the hippocampus was also observed in glioma patients using the dALFF. The dALFF, which is quantified as the variance in ALFF over time using sliding-window analysis, is a proxy for intrinsic brain activity [32]. Our results revealed a significant increase in neuronal activity in the bilateral hippocampus in high-grade glioma patients, which may indicate that more neuronal activity occurs, such as neurogenesis, in the hippocampus under the stimulation of malignant glioma. Importantly, our findings agree with previous results on glioma induced secondary neuronal reactions of the remote hippocampus in glioma rat model using PET [28].

The common reason for temporal lobe epilepsy is hippocampal sclerosis, which pathologically represents neuronal loss and gliosis [33]. TAE is a common symptom in glioma patients [34]. A recent study reported that the volumes of the caudate nucleus and thalamus ipsilateral to the tumor were significantly decreased in glioma patients with TAE [26]. Thus, we detected whether TAE affected hippocampal volume in glioma patients and found no statistically significant difference between patients with and without seizures. This result is similar to a recent study that investigated the alteration of hippocampal volume in glioma patients prior to radiotherapy [35]. IDH-mutation status, which represents slower local tumor growth rates and the less-infiltrative nature of diffuse tumor cell migration, has emerged as a major prognostic molecular marker in glioma patients [36–39]. Using DTI analysis, microstructural alterations in white matter distant from the tumor location in IDH-wildtype glioma patients compared with IDH-mutated glioma patients were observed, which may be attribute to tumor cell invasion with fiber compression and ensuing axonal damage [25]. In our sample, no difference in hippocampal volume was detected between IDH-wildtype and IDH-mutation glioma patients. This finding is likely related to the fact that the hippocampus is a brain region that is less prone to tumor invasion [27].

In the human brain, the hippocampus is the structure most susceptible to aging, and its GMV decreases in adults with advancing age [40–42]. In our study, we found that this phenomenon was destroyed by glioma, and aging patients had a large hippocampal volume distribution, especially in the HGG group. This finding also indicates that human hippocampal plasticity to pathological stimuli exists throughout life. A higher pathological grade often reflects more rapid tumor proliferation/infiltration, more serious damage to brain structure, and poorer prognosis [17, 43]. We observed that hippocampal volume and neuronal activity had stronger response in patients with high-grade glioma than in patients with low-grade glioma. A larger hippocampal volume in patients with a higher pathological grade may indicate that the hippocampus will appear to respond the same as severe pathological stimulation. Considering that the increase in hippocampal volume is a response of the brain to pathological stimulation, it may be related to malignant disease. In clinic, OS can more truly reflect tumor malignancy in individual patients. Thus, we further investigated whether there was a correlation between overall survival and hippocampal volume in high-grade glioma patients using Kaplan-Meier curves and log-rank tests. Surprisingly, our results showed that a large hippocampal volume was associated with poor overall survival. Furthermore, Cox proportional hazards regression was performed to investigate the predictive value of hippocampal volume, considering that histological grade (III, IV), age, IDH1 status, and contrast-enhancing volume are associated with OS in patients with high- grade glioma. These factors were also analyzed in Cox proportional hazards regression, and our results revealed that a large hippocampal volume was an independent factor for worse OS. These findings suggest that hippocampal volume may be used as an objective imaging marker to predict tumor malignancy in patients with high-grade glioma.

There are some limitations in the current study. The main limitation is the lack of histological studies to explain the physiological mechanism of hippocampal volume enlargement in glioma patients. In addition, our cohort was small in size and from a single institution. Larger data from other institutions will be needed to confirm and validate these findings.

## CONCLUSIONS

These important findings suggest that the hippocampus has a remarkable degree of plasticity in response to pathological stimulation in the glioma. The reaction of the hippocampus to glioma may be related to tumor malignancy.

## MATERIALS AND METHODS

### Participants

99 pathologically-confirmed glioma (WHO grades II, III, and IV) patients with no left or right hippocampus involvement, who accepted treatment in the Department of Neurosurgery, Beijing Tiantan Hospital, from September 2016 to December 2017, were enrolled in our study to investigate the structural alteration and neuronal reaction of the remote hippocampus in glioma. All subjects underwent anatomical MRI and resting-fMRI. Clinical variables including age, sex, education, tumor associated epilepsy (TAE), and pathological information, were obtained. Among these subjects, 52 patients had low-grade glioma (LGG group, left hemispheric glioma: n = 25, right hemispheric glioma: n = 27) and 47 patients had high-grade glioma (HGG group, left hemispheric glioma: n = 25, right hemispheric glioma: n = 22). The inclusion criteria for the patient group were 18–70 years of age and histologically proven glioma. The exclusion criteria were as follows: a history of stroke, cerebral trauma, brain surgery or brain radiotherapy, other intracranial abnormalities (i.e., arachnoid cysts), T1-weighted contrast enhancement (suggesting a tumor) or T2-weighted FLAIR hyperintensity (suggesting edema) extended to either the left or right hippocampus, bilateral extension of the lesion, an inability to complete the MRI examinations, or preprocessing issues (i.e., head motion). The healthy controls (HCs) were composed of 80 neurologically intact participants. Individuals with a history of neurodegenerative, neurodevelopmental, or psychiatric disease, substance use disorders involving alcohol or heroin, an inability to complete the MRI examinations, or preprocessing issues (i.e., head motion) were excluded.

In addition, 71 patients with meningioma and 73 HCs were also recruited in our study to assess whether other primary brain tumors affect hippocampal volume.

This study was approved by the medical ethics committee of Beijing Tiantan Hospital. Written informed consent was obtained from the legal representatives of all the patients and all the healthy volunteers, in accordance with the Declaration of Helsinki. The relevant guidelines and regulations were strictly observed in our experimental procedures.

### Image acquisition

All subjects were scanned on the 3.0 Tesla Siemens Prisma MRI scanner (Siemens Healthnieer, Erlangen, Germany) with a standard head coil prior to surgery. The 3D T1 weighted sagittal anatomical image was acquired (192 slices, slice thickness/gap = 1/0.5 mm, repetition time = 2530 ms, echo time = 2.55 ms, acquisition matrix = 512 × 512; flip angle = 12 deg, FOV = 256 × 256 mm^2^ with an in-plane resolution of 0.7 × 0.7 mm^2^). Resting state fMRI were acquired with an echo-planar image sequence (30 axial slices, slice thickness/gap = 5/0.5 mm, repetition time = 2000 ms, echo time = 30 ms, acquisition matrix = 64 × 64, 200 volumes, field of view (FOV) = 192 × 192 mm^2^, in-plane resolution = 3.0 × 3.0 mm^2^). During the resting state acquisition, all the participants were instructed to relax, stare at fixation point on the center screen and not to think of anything. The T2 image parameters included: repetition time = 5000 ms; echo time = 105 ms; flip angle = 150 degrees; 33 slices; field of view = 199 × 220 mm^2^; voxel size = 0.49 × 0.49 × 3.9 mm^3^; matrix = 406 × 448.

### Tumor masking

To define anatomical location of tumor in each patient, the tumor mask was traced slice by slice with MRIcron software (https://www.mccauslandcenter.sc.edu/crnl/tools) on T2 image for each patient. A volume of interest (VOI) was created was generated with MRIcron for each patient. Then T2 image and VOI were registered to the Montreal Neurological Institute template using the standard nonlinear spatial normalization algorithm provided by SPM12. Finally, all tumor masks were overlapped in Ch2bet template.

### Contrast-enhanced volume

Postcontrast T1-weighted images (TE 15 ms, TR 450 ms, and slice thickness 5 mm) were acquired on 3.0 Tesla Siemens or GE scanner after injection of gadopentetate dimeglumine (Ga-DTPA Injection, Beilu Pharma, Beijing, China) at a dose of 0.1 mmol/kg. The contrast-enhanced volume of the glioma, including areas of contrast enhancement and areas of central necrosis, was measured on postcontrast T1-weighted images [44]. Contrast enhancement of the tumor was traced with MRIcron software (https://www.mccauslandcenter.sc.edu/crnl/tools) by two neurosurgeons.

### Immunohistochemistry for IDH1-R132H

In the current study, the IDH1 mutation status was routinely evaluated by experienced pathologists using typical tumor samples collected from the glioma patients. Immunostaining was performed in accordance with the manufacturer’s protocol. IDH1 immunostaining was performed on an automated immunohistochemistry system (BenchMark ULTRA, Ventana Medical Systems, Strasbourg, France). Anti-human IDH1-R132H antibodies were purchased from Zhongshan Golden Bridge Biotechnology (Beijing, China). For IDH1-R312H staining, a strong cytoplasmic immunoreaction product was scored as positive, and weak, diffuse staining and macrophages staining were scored as negative [45].

### Data preprocessing

Structural image preprocessing was conducted using statistical Parametric Mapping toolbox (SPM12, http://www.fil.ion.ucl.ac.uk/spm) and the CAT12 toolbox (http://www.neuro.uni-jena.de/cat/) in the MATLAB environment (MATHWORKS, California, USA). In summary, 3D T1 images were manually reoriented and shifted to define the anterior commissure as the origin (mm coordinate 0, 0, 0). Then, using the module “Segment Data” of CAT12, 3D T1-weighted images were segmented into gray matter (GM), white matter (WM), and CSF. Furthermore, neuromorphometric atlases was selected for region of interest (ROI) analysis. The modulated warped GM images were then normalized to Montreal Neurological Institute (MNI)-152 standard space with an isotropic voxel resolution of 1.5 mm × 1.5 mm × 1.5 mm. Next, using “Display one slice for all images”, the data quality was checked after the segmentation and normalization procedures. In addition, the total intracranial volume (TIV) for each subject was calculated with the “Estimate TIV” module. Using a boxplot and correlation matrices, sample homogeneity was checked to identify outliers by visualizing the correlation between the volumes. Finally, each individual’s modulated GM map was smoothed with an 8-mm full-width at the half-maximum Gaussian kernel.

The rs-fMRI data were preprocessed using the SPM12 and Data Processing & Analysis of Brain Imaging toolbox (DPABI, http://www.restfmri.net/) [46]. To allow the signal to reach equilibrium and the subjects to adapt to the environment, the first 10 volumes from each participant were deleted. Realignment was conducted to correct head motion. Participants with a head motion >2.5 mm in maximum displacement or >2.5 rotation in angular motion were excluded from the study. The mean frame displacement (FD) was calculated for each subject according to a previously published formula [47]. In addition, each subject’s mean FD was included in all group-level analyses as a covariate to further control the effect of head movement. After the motion correction, the 3D T1 images were coregistered to the mean functional image of each participant. The coregistered 3D T1 images were then segmented as GM, WM, and CSF, followed by normalization to MNI space using the Diffeomorphic Anatomical Registration Through Exponentiated Lie algebra (DARTEL) algorithm [48]. Next, the motion-corrected functional volumes were also normalized to the MNI space using the normalization parameters for their respective structure images and resampled into a voxel size of 3 mm × 3 mm × 3 mm. Subsequently, several spurious variances, including Friston 24 head motion parameters, CSF, WM signals, and linear trends were regressed out.

### Voxel-based morphometry (VBM) analysis

After data processing, we assessed the difference in gray matter volume (GMV) between the patient groups (left HGG, left LGG, right HGG, right LGG) and HCs with two sample t-test in cat 12 with TIV, education, age and sex as covariates. In addition, we applied absolute masking with a threshold of 0.2 and proportional scaling for global normalization referenced in a previous study [49]. Next, bilateral hippocampal region of interest (ROI) masks using the WFU Pickatlas tool box based on the Anatomical Automatic Labeling (AAL) template was applied to explore the structural changes in the hippocampus. The false discovery rate (FDR) was used for correction with p < 0.05 at the cluster level and cluster sizes of k > 20.

### ROI-based hippocampal volume

We also tested the left and right hippocampal volume. Using “extract ROI data”, the GMV from each region, including the hippocampus, was extracted according to the Neuromorphometric atlases (http://www.neuromorphometrics.com/).

### Dynamic amplitude of low-frequency fluctuation (dALFF) analysis

Temporal dynamic analysis (TDA) toolkits based on DPABI sliding window-based analyses was applied to examine the dALFF variability over the whole brain [50]. In the sliding window analysis, ALFF are calculated in a temporal window of a certain size and shape. In our study, a window length of 40 TRs (80 s) and a shifting step size of one TR (2 s) wer used to calculate the temporal variability of the dALFF. The time series comprised 190 time points after removing the first 10 time points, which were segmented into 151 windows for each individual. For each sliding window, a ALFF map was obtained. To investigate the temporal variability of brain activity, the standard deviation (SD) of ALFF values at each voxel across 151 windows was calculated. To reduce the global effects of variability across subjects, the dALFF of each voxel was divided by the global mean dALFF within a gray matter mask. Finally, the mean normalized dALFF maps were spatially smoothed with an isotropic Gaussian kernel of 4 mm full-width-at-half maximum (FWHM). Bilateral hippocampal ROI masks using the WFU Pickatlas tool box based on the AAL template was applied to explore neuronal reactions in the hippocampus. The FDR was used to corrected with p < 0.05 at the cluster level and cluster sizes of k > 10. Then, the left/right hippocampal ROI masks based on the AAL template were applied to extract the value of dALFF in all subjects to assess the neuronal activity of the entire unilateral hippocampus.

### Statistical analyses

Statistical analyses for the data extracted from imaging and non-imaging data were performed with the Statistical Package for Social Science (SPSS) (SPSS Inc., version 25.0, Chicago, IL), with a two-tailed significance level of p < 0.05. One-way ANOVA with Bonferroni correction was used to assess the differences in age, education, TIV, hippocampal volume and dALFF among the HGG, LGG, and HCs groups. The correlations between hippocampal volume and age were assessed by Pearson’s correlation analysis. Two-sample t-test were used to analyze the effect of TAE on hippocampal volume between patients with and without TAE. To analyze the effect of tumor on ipsilateral and contralesional hippocampal volume, the difference between right and left hippocampal volume was calculated in all subjects; then, one-way ANOVA with Bonferroni correction was used to assess the differences among HGG, LGG, and HCs groups. For high-grade glioma patients, we detected differences in hippocampal volume between IDH-mutated (IDHmut) and wild-type patients (IDHwt) using two-sample t-test. Survival analysis including Kaplan-Meier curves with log-rank tests and Cox multivariate logistic regression analyses were performed to assess the relationship between hippocampal volume and overall survival in high-grade glioma patients.

### Availability of data

The datasets generated for this study are available on request to the corresponding author

## Supplementary Material

Supplementary Table 1
