# Supplementary material for: Structural plasticity of the bilateral hippocampus in glioma patients
Source: Aging (Albany NY). 2020 Jun 5;12(11):10259–74. doi: 10.18632/aging.103212 (PMC7346025; doi:10.18632/aging.103212)
Supplement: Supplementary Table 1 [file aging-12-103212-s001..pdf]

SUPPLEMENTARY TABLE

Supplementary Table 1. VBM analyses for glioma patient groups compared with HCs.

Analysis	Group	Cluster size (in voxels)	Peak MNI coordinate			Peak level T value
			X	Y	Z	
VBM	Left HGG vs HCs	175	-14	-40	10	4.03
			-20	-33	0	3.15
	Left LGG vs HCs	20	-14	-39	10	3.53
		74	-34	-28	-6	3.07
	Right HGG vs HCs	473	14	-38	10	3.89
			26	-33	8	3.66

T values were determined by a 2-sample t test (Cluster level $p < 0.05$, FDR corrected), Voxel size = $1.5 \times 1.5 \times 1.5$ mm^3.
